# RNA‐binding protein HuR suppresses senescence through Atg7 mediated autophagy activation in diabetic intervertebral disc degeneration

**DOI:** 10.1111/cpr.12975

**Published:** 2020-12-28

**Authors:** Zhenxuan Shao, Libin Ni, Sunli Hu, Tianzhen Xu, Zaher Meftah, Zupo Yu, Naifeng Tian, Yaosen Wu, Liaojun Sun, Aimin Wu, Zongyou Pan, Linwei Chen, Weiyang Gao, Yifei Zhou, Xiaolei Zhang, Xiangyang Wang

**Affiliations:** ^1^ Department of Orthopaedics The Second Affiliated Hospital and Yuying Children’s Hospital of Wenzhou Medical University Wenzhou China; ^2^ Key Laboratory of Orthopaedics of Zhejiang Province Wenzhou China; ^3^ The Second School of Medicine Wenzhou Medical University Wenzhou China; ^4^ Department of Orthopaedics Zhuji People's Hospital of Zhejiang Province China; ^5^ Department of Orthopaedics The Second Affiliated Hospital of Zhejiang University School of Medicine Hangzhou China

**Keywords:** Atg7, autophagy, diabetes, HuR, intervertebral disc degeneration, senescence

## Abstract

**Objectives:**

Diabetes is a risk factor for intervertebral disc degeneration (IVDD). Studies have demonstrated that diabetes may affect IVDD through transcriptional regulation; however, whether post‐transcriptional regulation is involved in diabetic IVDD (DB‐IVDD) is still unknown. This study was performed to illustrate the role of HuR, an RNA‐binding protein, in DB‐IVDD development and its mechanism.

**Materials and Methods:**

The expression of HuR was evaluated in nucleus pulposus (NP) tissues from diabetic IVDD patients and in high glucose‐treated NP cells. Senescence and autophagy were assessed in HuR over‐expressing and downregulation NP cells. The mRNAs that were regulated by HuR were screened, and immunoprecipitation was applied to confirm the regulation of HuR on targeted mRNAs.

**Results:**

The results showed that the expression of HuR was decreased in diabetic NP tissues and high glucose‐treated NP cells. Downregulation of HuR may lead to increased senescence in high glucose‐treated NP cells, while autophagy activation attenuates senescence in HuR deficient NP cells. Mechanistic study showed that HuR prompted Atg7 mRNA stability *via* binding to the AU‐rich elements. Furthermore, overexpression of Atg7, but not HuR, may ameliorate DB‐IVDD in rats in vivo.

**Conclusions:**

In conclusion, HuR may suppress senescence through autophagy activation *via* stabilizing Atg7 in diabetic NP cells; while Atg7, but not HuR, may serve as a potential therapeutic target for DB‐IVDD.

## Graphical abstract

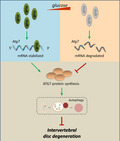

## 1 INTRODUCTION

Low back pain is a common symptom of various medical conditions, and up to 80% of the population may suffer from it at different stages of life.^1^ Patients can be paralysed and incapable to work due to severe low back pain, which places a severe burden on their lives and socio‐economic development.^2^ Studies demonstrated that over 40% of low back pain is caused by intervertebral disc degeneration (IVDD).^3^, ^4^, ^5^ Nevertheless, the specific pathological mechanism underlying IVDD has not been fully elucidated.

Pathological factors including inflammation, abnormal mechanical stress, ageing, obesity and smoking are related to IVDD development.^6^ Diabetes is a newly discovered risk factor for IVDD with increasing concerns.^7^, ^8^ Diabetes is a metabolic disorder considered by high blood glucose level over a long period. Epidemiological studies have implied that diabetes is an important risk factor for IVDD,^7^ and animal study has shown that diabetes accelerates the progress of IVDD.^9^ However, the mechanism of diabetic intervertebral disc degeneration (DB‐IVDD) remains unclear.

Intervertebral disc is mainly made up of three types of cells: the gelatinous internal NP cells, the external annulus fibrosus cells, and endplate chondrocytes in the lower and upper endplates.^10^ Gelatinous NP allows the vertebral disc to bear diverse mechanical pressures from various activities. The abnormal function of NP cells is considered as the initiating factor of IVDD. Accumulating evidence demonstrated that premature senescence of nucleus pulposus (NP) cells is one of the main pathogenic mechanisms of IVDD. Animal study has demonstrated that diabetes may promote senescence in NP cells.^8^ In vitro study showed that high glucose may induce senescence in NP cells.^11^, ^12^ Moreover, eliminating senescent NP cells are regarded as an efficacious therapeutic strategy for IVDD.^13^, ^14^ However, how diabetes may induce senescence in NP cells is still not clear.

Autophagy is a process that degrading and recycling cellular component, and it is an important cyto‐protective mechanism for intracellular homeostasis maintenance.^15^ Autophagy has been shown to protect against premature senescence in various conditions,^16^, ^17^ while autophagy deficient may lead to senescence.^18^ Aberrant autophagy is related to various degenerative diseases, such as Parkinson's disease,^19^ osteoarthritis,^20^ Alzheimer's disease,^21^ as well as IVDD.^22^ Therefore, we assume that autophagy may play a role in the pathogenesis of DB‐IVDD.

Autophagy is regulated at transcriptional, post‐transcriptional, translational and post‐translational levels.^23^ It has been demonstrated by our group that the transcription factor BRD4 is abnormally highly expressed in diabetic nucleus pulposus tissue, and inhibiting BRD4 expression can postpone the progress of DB‐IVDD *via* autophagy activation ^24^; we also found that prompting the transcription factor TFEB can also inhibit premature senescence in NP cells *via* autophagy activation.^25^ These studies suggest that dysfunction of autophagy may occur at transcriptional level; however, whether the post‐transcriptional level is involved is still not clear.

Post‐transcriptional regulation is the control of gene expression at the RNA level, including the regulation of RNA editing, RNA stabilization, RNA splicing, RNA cleavage and translation. HuR (Human antigen R, encoded by Embryonic Lethal Abnormal Vision‐Like Protein 1, ELAVL1) is an RNA‐binding protein,^26^ it may effect the stabilization of mRNAs so as to regulate their expression and is involved in various biological progresses. Recent studies showed that HuR is related to autophagy ^26^; also, HuR is regarded as a crucial metabolic regulator of diabetes,^27^ which implied that HuR may play a role in DB‐IVDD *via* autophagy regulation.

In the current study, we found that HuR downregulation prompts senescence through autophagy inactivation in high glucose‐treated NP cells, and HuR regulates Atg7 expression through adjusting its mRNA stability. In addition, we found that Atg7 overexpression inhibits senescence in high glucose‐treated NP cells, and ameliorated the progress of DB‐IVDD in vivo.

## 2 MATERIAL AND METHODS

### 2.1 Ethics statement

All treatments on animals and animal care procedures after operation were strictly achieved in keeping with the guidelines for Animal Care and Use outlined by the Committee of Wenzhou Medical University (WYDW2020‐0160). Human tissue collection and treatments were also permitted by the Second Affiliated Hospital and Yuying Children's Hospital of Wenzhou Medical University Ethics Committee (LCKY2020‐86), following the guidelines of the Helsinki Declaration.

### 2.2 Reagents and antibodies

Glucose, streptozotocin (STZ), citrate buffer, dimethylsulphoxide (DMSO) and collagenase type II were purchased from Sigma‐Aldrich. Rapamycin was acquired from MedChemExpress LLC. Cell culture reagents were obtained from Gibco. Cell counting kit‐8 (CCK‐8) was purchased from Dojindo. Glucometer was obtained from Yuwell. The primary antibody against GAPDH (60004‐1‐Ig) was purchased from Proteintech, primary antibodies against p21WAF1 (ab86696), p62 (ab240635), LC3 (ab62721), and Atg7 (ab223365) were acquired from Abcam, and primary antibodies against HuR (#12582), p53 (#2524), p‐p53 (#9284), p16INK4A (#80772) and LC3‐II (#2775) were purchased from Cell Signaling Technology. Goat anti‐rabbit and anti‐mouse IgG‐HRP antibodies were purchased from Bioworld. In Jackson ImmunoResearch, Alexa Fluor^®^ 594‐ and 488‐conjugated secondary antibodies were obtained. In Beyotime, 4′,6‐Diamidino‐2‐phenylindole (DAPI) was purchased.

### 2.3 Extraction and culture of NP cells

From healthy NP tissues of young Sprague‐Dawley rats (Male, 100‐150 g, 4 week), NP cells were extracted. NP tissues were cut up into 1 mm^3^ and washed with phosphate‐buffered saline (PBS). Then, the NP tissues were digested of 0.25% collagenase type II at 37°C for 2 hours. After centrifugation, the cells were cultured in DMEM/F12 culture medium (Gibco, Invitrogen, Grand Island, NY) with 1% antibiotics and 15% foetal bovine serum (FBS; Gibco), in an incubator with 5% CO_2_ at 37°C. Human NP tissues were isolated from normal and diabetic patients to compare the HuR expression by immunofluorescence and Western blot analysis.

### 2.4 Lentivirus transfection

The NP cells reaching 40‐60% confluence were transfected using lentivirus (LV‐siHuR was from Cell‐land, China, TACCAGTTTCAATGGTCATAA; LV‐HuR was from OBiO, China, NM_001108848; and LV‐Atg7 was from GeneChem, China, NM_001012097) at a multiplicity of infection (MOI) of 50.

### 2.5 STZ‐induced diabetic model and surgical procedure

Adult male Sprague‐Dawley rats (200‐250 g, 8 weeks old, n = 32) were purchased from the Experimental Animal Institute of Wenzhou Medical University. All the rats were randomly divided into four groups: control group, diabetes group, LV‐Atg7 group and LV‐HuR group.

According to the previous study, the rats of the diabetes, LV‐ Atg7 and LV‐HuR groups were intraperitoneally injected with STZ at 65 mg/kg body weight in 0.1 mol/l citrate buffer, and the rats were fed on high‐fat diet. Then, blood glucose levels were examined by a glucometer (Yuwell Co., China) at 3 days after STZ treatment, then examined again at 5 days after STZ treatment. A blood glucose level greater than 16.7 mM was defined as successful diabetes.^8^

Then, the rats were anaesthetized with 2% (w/v) pentobarbital (40 mg/kg) using intraperitoneal injection. And 27‐G needles were used to puncture the whole layer of annulus fibrosus for 1 minute.^28^

### 2.6 X‐ray image acquisition and magnetic resonance imaging (MRI)

The rats were postured in a prone position, and the images were captured by the X‐ray irradiation system (Kubtec, USA). The coccyx of the rats was analysed in sagittal T2‐weighted images using a 3.0 T clinical magnet (Philips Intera Achieva 3.0 MR).^25^

### 2.7 Statistical analysis

Data are presented as the means ± standard deviation (SD). The data were analysed *via* Graphpad Prism (one‐way analysis of variance (ANOVA) and Tukey's post hoc test). Non‐parametric data (Pfirrmann scores and histological grades) were analysed by the Kruskal‐Wallis H test. *P* values <.05 were considered as statistically significant.

## 3 RESULTS

### 3.1 The expression of HuR is decreased in diabetic NP tissues and high glucose‐treated NP cells

**FIGURE 1:**
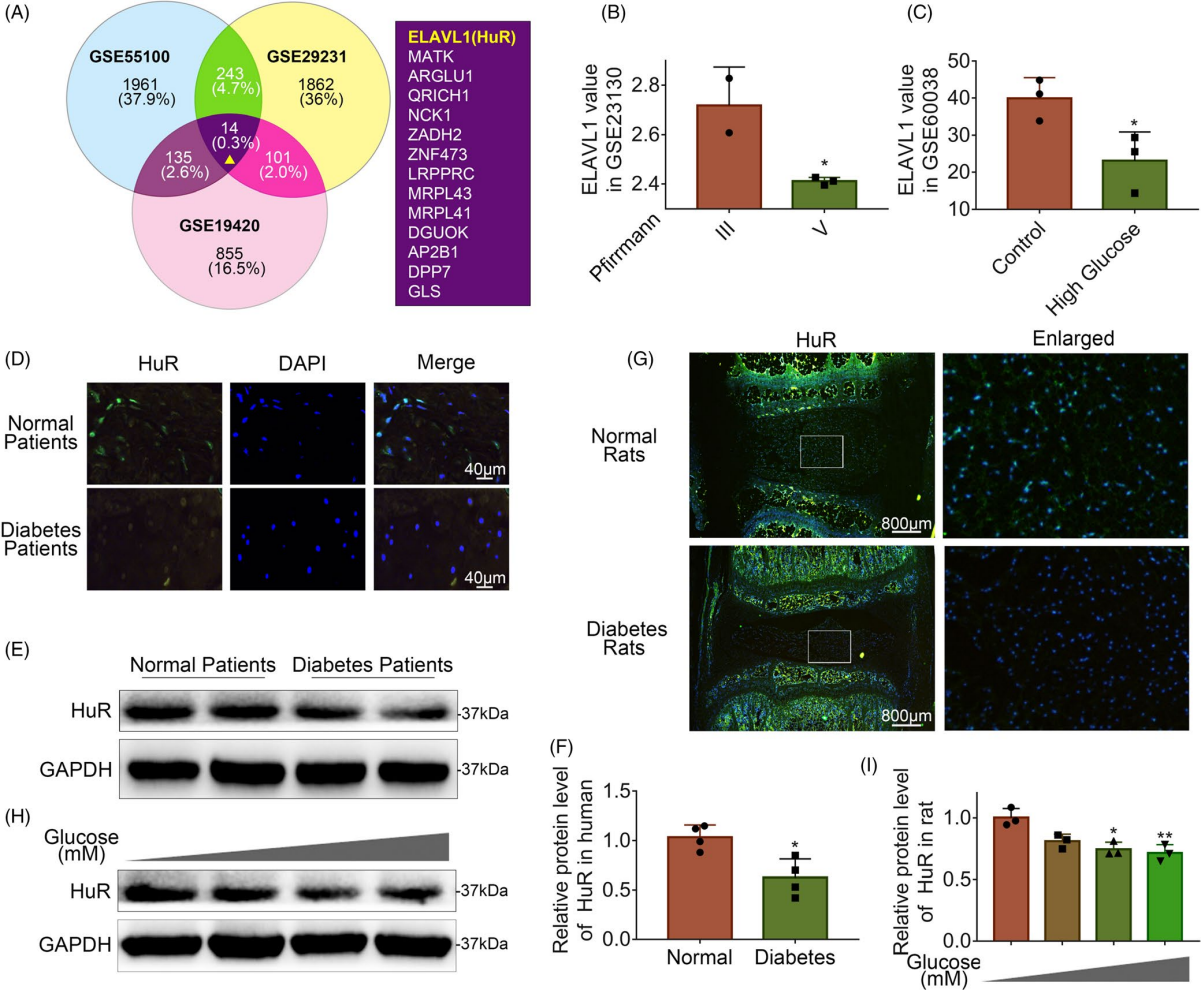
The expression of HuR is decreased in diabetic NP tissues and high glucose‐treated NP cells. A, Venn diagram of all differentially down‐regulated genes, and data of three diabetic tissues from GEO DataSets (Series Accession: GSE55100, GSE29231 and GSE19420). B, The expression of HuR gene of degenerated NP tissues (Series Accession: GS23130). C, The expression of HuR gene of high‐glucose treatment cells (Series Accession: GSE60038). D, Representative immunofluorescence staining of HuR in NP tissue from normal and diabetic patients (scale bar: 40 μm). E, F, The protein expression of HuR in NP tissue from normal and diabetic patients, detected by Western blot and quantified by Image J software. G, Representative immunofluorescence staining of HuR in NP tissues from normal and diabetes rats (scale bar: 800 μm). H, I, The protein expression of HuR in rat NP cells after various concentrations of glucose treatment for 6 hours, detected by Western blot. All data were shown as mean ± SD. *P < .05, **P < .01

Post‐transcriptional regulation is the control of gene expression at the RNA level, which contributes substantially to gene expression regulation across tissues. In order to explore the post‐transcriptional regulators that are involved in the process of DB‐IVDD, we screened data from the GEO DataSets (https://www.ncbi.nlm.nih.gov/gds). Three series of diabetic data (Series Accession: GSE55100, GSE29231 and GSE19420) are included into the analysis, and the Venn diagram showed that ELAVL1(HuR) was one of commonly down‐regulated genes during DB‐IVDD (Figure 1A, Figure S1). And it was found from dataset GSE60038 that ELAVL1(HuR) gene level was also depressed in NP cells under high‐glucose stimulation (Figure 1C). Interestingly, it was found from dataset GSE23130 that ELAVL1(HuR) gene level was also depressed during IVDD (Figure 1B). Thus, data from GEO DataSets indicate that there may be a connection between HuR and DB‐IVDD.

To further certify that ELAVL1 (HuR) expression is decreased in IVDD, we collected NP tissues from normal and diabetic IVDD patients to detect HuR expression by immunofluorescence and Western blot. The immunofluorescence results showed that the expression of HuR decreased in diabetic NP tissues (Figure 1D); the Western blot analysis showed that the expression of HuR in diabetic patients was lower than that in the normal individuals (Figure 1E‐F). Meanwhile, we established a rat model to examine the HuR expression in diabetic rats by immunofluorescence. The results showed the expression of HuR was depressed in diabetic rat NP tissues (Figure 1G). The above result shows that the expression of HuR decreases in both human diabetic NP tissues and rat diabetic NP tissues.

Further, we isolated NP cells from normal rat NP tissues and treated them with ascending concentrations of glucose (5, 50, 100 and 200 mM) for 6 hours. The Western blot analysis showed that the expression of HuR was decreased in a dose‐dependent manner (Figure 1H‐I).

### 3.2 HuR downregulation prompts senescence in high glucose‐treated NP cells

**FIGURE 2:**
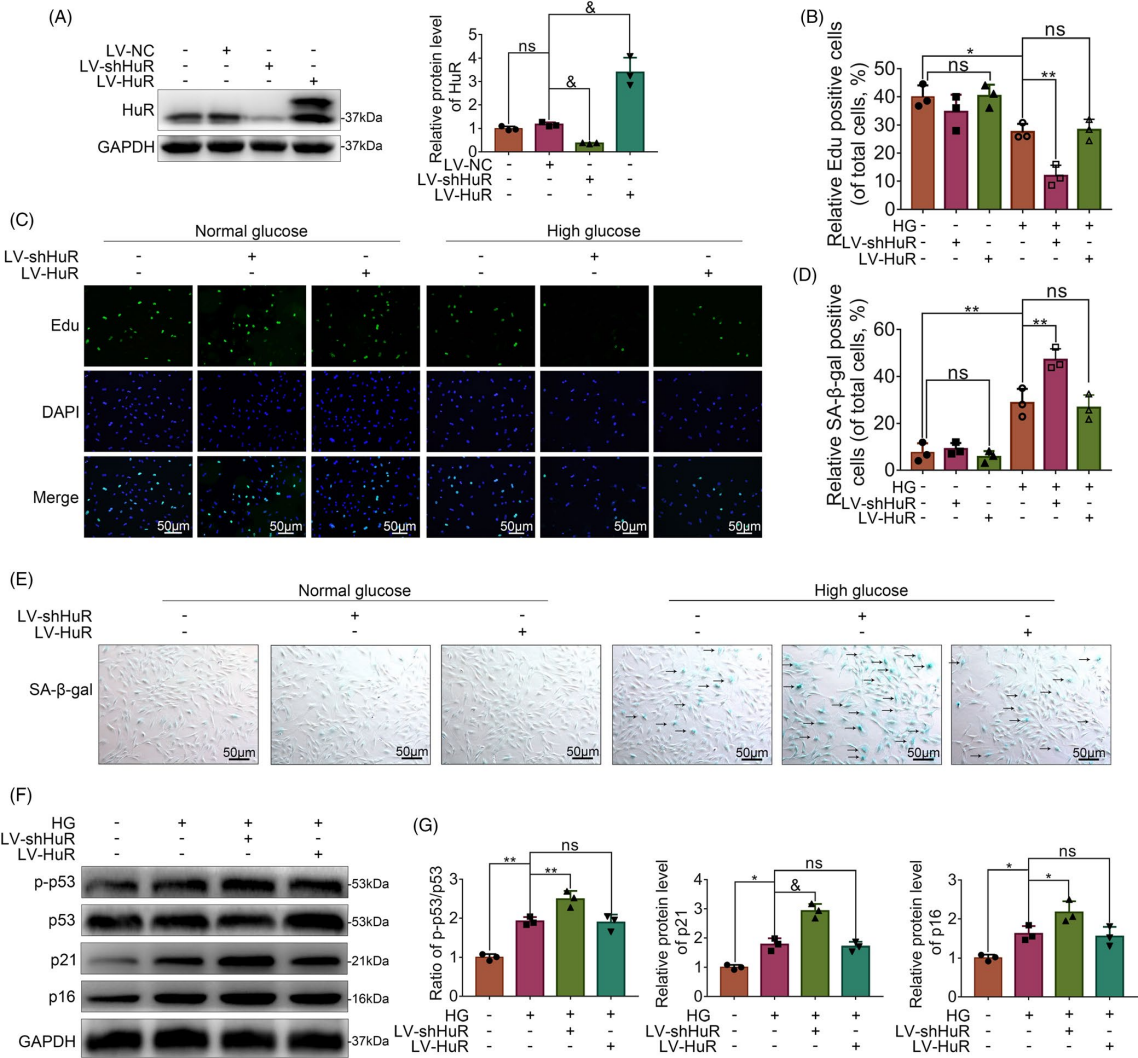
HuR downregulation prompts senescence in high glucose‐treated NP cells. The cells were transfected with LV‐NC, LV‐shHuR or LV‐HuR before high‐glucose treatment (100 mM). A, The protein expression of HuR in rat NP cells transfected with lentivirus, detected by Western blot. B, C, EdU staining for NP cells with or without high‐glucose treatment for 6 hours and transfected with lentivirus (scale bar: 50 μm). D, E, SA‐β‐gal staining for NP cells (scale bar: 50 μm). F, G, The protein expression of p21 and p16INK4a, and the ratio of p‐p53/p53 were detected by Western blot and quantified by Image J software. All data were shown as mean ± SD. *P < .05, **P < .01, &*P* < .001

To explore the effects of HuR on DB‐IVDD in vitro, we manipulated HuR expression in NP cells by lentivirus‐shHuR (LV‐shHuR) and lentivirus‐HuR (LV‐HuR) transfection. The lentivirus transfection efficiency was verified by Western blot (Figure 2A).

Next, we analysed the EdU activity, SA‐β‐gal activity and the expressions of p‐p53, p53, p21 and p16INK4a expression in NP cells, which are usually used as indicators of senescence. The EdU assay showed that without high‐glucose treatment, there was no significant difference in the LV‐NC, LV‐shHuR and LV‐HuR groups; however, the proliferative activity of NP cells was expectedly decreased under high‐glucose treatment, while more decreased proliferative activity was found in the LV‐shHuR group (Figure 2B, C). Meanwhile, SA‐β‐gal assay revealed that without high‐glucose treatment, there was no significant difference among groups; under high‐glucose treatment, HuR knockdown prompted SA‐β‐gal activity (Figure 2D, E). The Western blot analysis also showed that there were significantly higher ratio of p‐p53/p53, and higher levels of p21 and p16INK4a in high glucose‐treated rat NP cells with HuR knockdown (Figure 2F, G; Figure S2). These results indicate that the HuR downregulation prompts cellular senescence in high glucose‐treated rat NP cells.

To our surprise, we found that HuR upregulation could not reversed increased senescence in high glucose‐treated rat NP cells. The phenomenon was addressed in the discussion.

### 3.3 HuR regulates autophagy in high glucose‐treated NP cells

In order to explore the working mechanism of HuR on DB‐IVDD in NP cells, we analysed genes that have been reported to be regulated by HuR in previous studies.^29^ The data from Mukherjee‘s research were included into KEGG pathway analysis. The result showed that various pathways were involved, among which autophagy signalling pathway is most likely to participate in the regulatory effects of HuR on senescence in DB‐IVDD (Figure S3).

**FIGURE 3:**
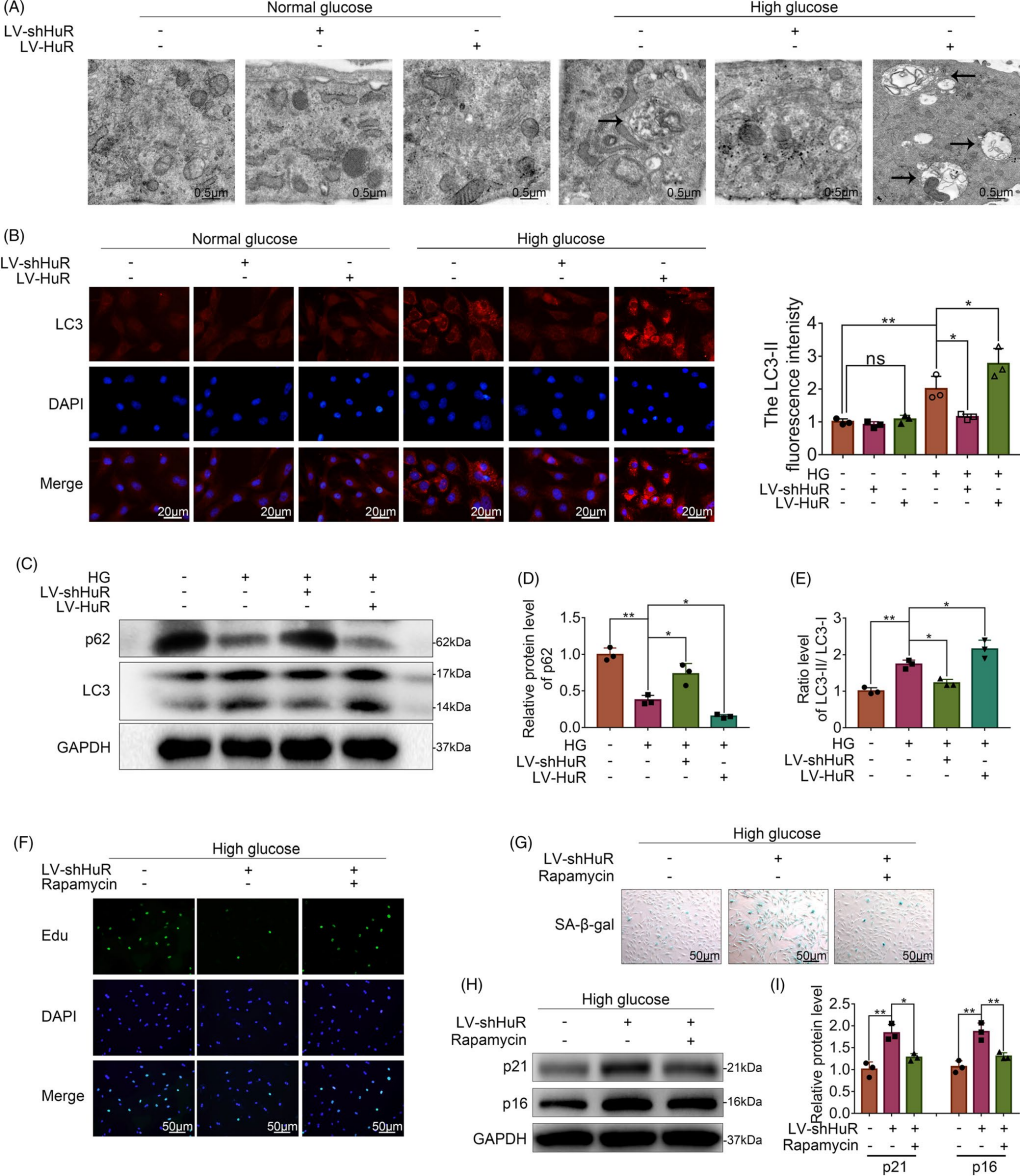
HuR regulates autophagy activation in high glucose‐treated NP cells. The cells were transfected with LV‐NC or LV‐shHuR or LV‐HuR before high‐glucose treatment (100 mM). A, TEM images of autophagic vesicles in rat NP cells (bar: 0.5 μm). B, The LC3‐II was detected by immunofluorescence combined with DAPI staining for nuclei (scale bar: 20 μm). C‐E, The protein expression of p62 and the ratio of LC3‐II/ LC3‐I in NP cells treated with or without high‐glucose treatment (100 mM) for 6 hours and transfected with lentivirus, detected by Western blot and quantified by Image J software. F, EdU staining for NP cells (scale bar: 50 μm). G, SA‐β‐gal staining for NP cells (scale bar: 50 μm). H, I, The protein expression of p21 and p16INK4a in NP cells with or without rapamycin (the classical autophagy activator, 10 μM, 24 hours), detected by Western blot. All data were shown as mean ± SD. *P < .05, **P < .01

To further exemplify the relationship between HuR activity and autophagy, we carried out the following experiments. Transmission electron microscopy (TEM) showed that without high‐glucose treatment, the number of autophagosome vacuoles was small in the LV‐NC, the LV‐shHuR and the LV‐HuR NP cells; under high‐glucose treatment, the number of autophagosome vacuoles increased significantly in the LV‐NC NP cells, while such a change was attenuated in LV‐shHuR NP cells, and the number of autophagosome vacuoles increased in LV‐HuR NP cells (Figure 3A).

The immunofluorescence results showed that without high‐glucose treatment, there was no significant difference among the LV‐NC, the LV‐shHuR and the LV‐HuR groups; under high‐glucose treatment, the LC3‐II fluorescence intensity was expectedly increased, while such a change was reversed in the LV‐shHuR group, increased in LV‐HuR NP cells (Figure 3B). In addition, we evaluated the level of autophagy by Western blot analysis. Results showed that autophagy dramatically decreased when HuR was down‐regulated, whereas autophagy dramatically increased when HuR was upregulated, as indicated by the LC3‐II/ LC3‐I ratio and the p62 expression (Figure 3C‐E, Figure S4).

These results indicate that HuR may not regulate autophagy in normal glucose condition; however, HuR may promote autophagy in high‐glucose condition.

### 3.4 HuR downregulation prompts senescence through autophagy inactivation in high glucose‐treated NP cells

To explore whether autophagy is related to the effect of HuR downregulation on senescence, NP cells were pre‐treated with a classical autophagy activator (rapamycin, 24 hours,10 μM), followed by Western blot, EdU and SA‐β‐gal analysis.

The EdU assay showed that proliferative activity of NP cells decreased by HuR downregulation, while rapamycin increased their proliferative activity (Figure 3F); HuR downregulation also prompted SA‐β‐gal activity, whereas rapamycin inhibited the SA‐β‐gal activity (Figure 3G). The Western blot results showed that significantly higher levels of p21 and p16INK4a induced by HuR downregulation, while such an upregulation was attenuated by rapamycin (Figure 3H‐I).

### 3.5 HuR downregulation inhibits autophagy via Atg7 in high glucose‐treated NP cells

**FIGURE 4:**
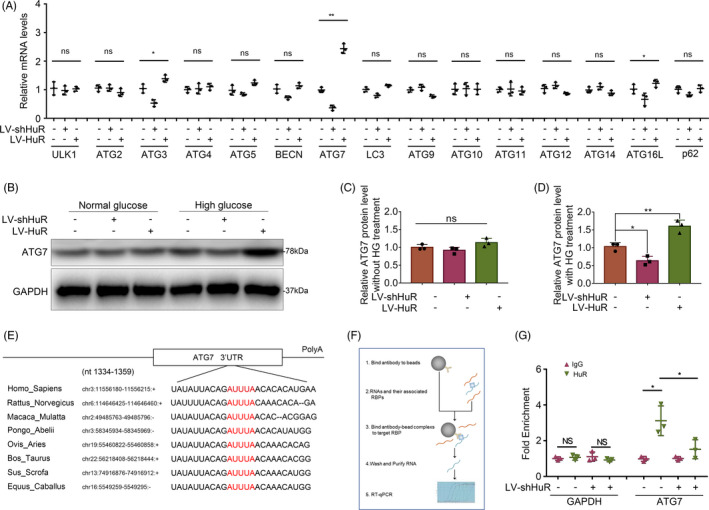
HuR regulates Atg7 expression through binding to Atg7 AU‐rich element and upregulating its mRNA level. The cells were transfected with LV‐shHuR or LV‐HuR before high‐glucose treatment (100 mM). A, Multiple autophagy‐related mRNA expressions in NP cells with high‐glucose treatment, detected by qPCR. B‐D, The protein expression of Atg7 in NP cells with or without high‐glucose treatment (100 μM) for 6 hours, detected by Western blot and quantified by Image J software. E, Potential HuR‐binding element at 3′UTR of Atg7 mRNA on multiple species. F, Schematic diagram showing the workflow of the RNA‐binding protein immunoprecipitation (RIP) assay. G, The mRNA expression of Atg7 in rat NP cells, detected by qPCR, and normalized to IgG isotype controls. All data were shown as mean ± SD. *P < .05, **P < .01

To explore the underlying mechanism between autophagy and HuR in high glucose‐treated NP cells, we measured the levels of multiple autophagy‐related genes by qPCR. The results showed that Atg3, Atg7 and Atg16 genes were differentially expressed when HuR was overexpressed or knocked‐down, while the change of Atg7 was the most significant (Figure 4A).

Further, we evaluated the expression of Atg7 with high‐glucose treatment by Western blot analysis and found that it was prompted in the NP cells with HuR upregulation, whereas the expression of Atg7 was depressed when HuR was down‐regulated (Figure 4B‐C). In addition, no significant difference was found in the expression of Atg7 in normal glucose treatment condition (Figure 4B, D). The Western blot showed that the expressions of Atg3 and Atg16 were not obviously changed in HuR‐regulated NP cells, compared to the LV‐NC NP cells (Figure S5). Our data indicate that Atg7 may be the key protein of HuR‐regulated autophagy in high glucose‐treated NP cells.

### 3.6 HuR regulates Atg7 expression through binding to Atg7 AU‐rich element and upregulating its mRNA level

Studies showed that HuR appears to regulate gene expression *via* stabilizing mRNAs and play roles in a variety of biological progresses.^30^ Bioinformatic study implied that there was a stable HuR‐binding element in the 3′UTR of Atg7 mRNA on multiple species (Figure 4E).

RNA‐binding protein immunoprecipitation (RIP) is a method used to recognize specific mRNA, which can bind to known protein; the workflow of RIP is shown in Figure 4F. Compared with immunoglobulin G (IgG)–combined RNA, HuR was significantly enriched for Atg7 (Figure 4G, IgG *vs* HuR); Compared with the negative control (GAPDH), HuR protein directly bound to Atg7 mRNA (Figure 4G, GAPDH *vs* Atg7). Meanwhile, the mRNA level of Atg7 decreased in HuR‐downregulated NP cells, which implied the mRNA level of Atg7 was also regulated by HuR (Figure 4G, LV‐NC *vs* LV‐shHuR).

### 3.7 Atg7 reverses HuR‐down‐regulated autophagy in high glucose‐treated NP cells

**FIGURE 5:**
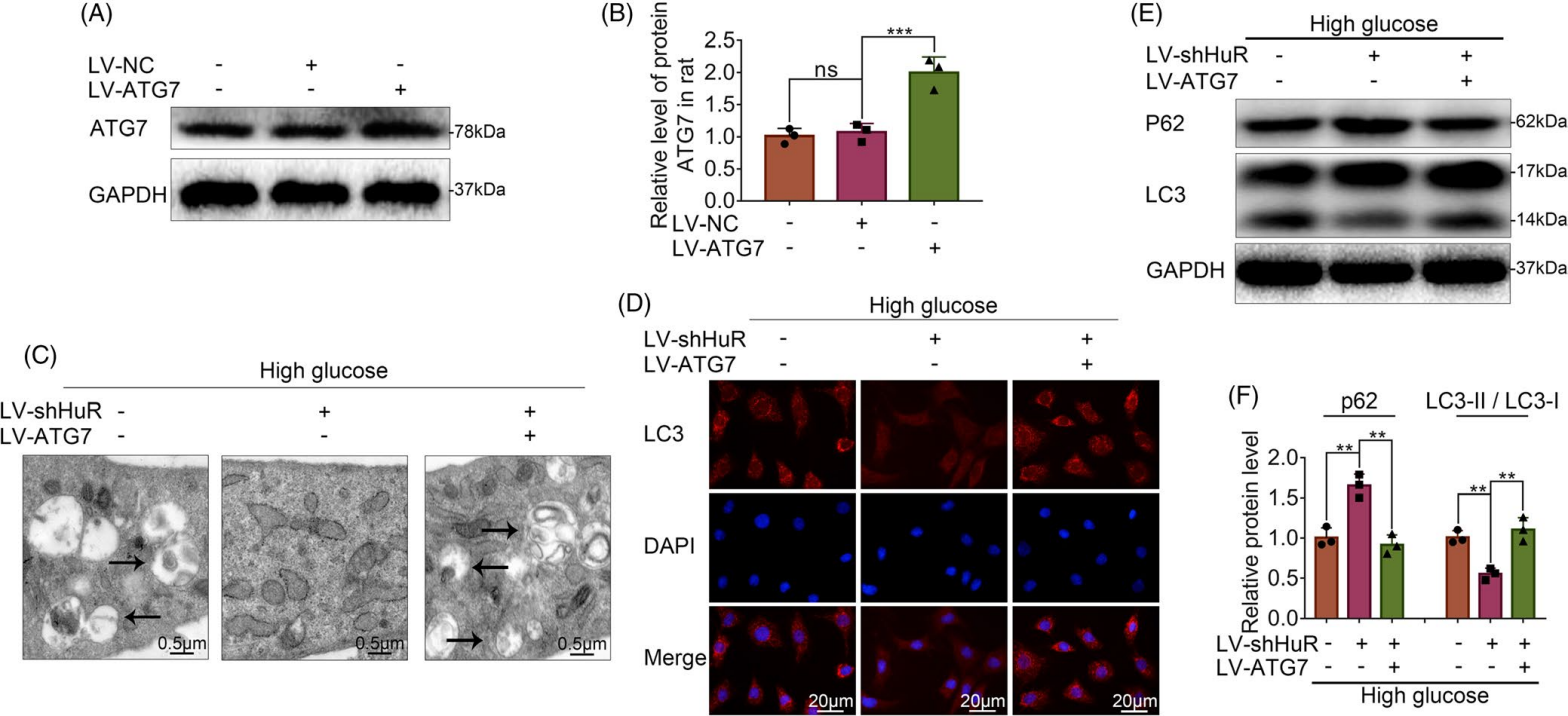
Atg7 reverses HuR‐down‐regulated autophagy in high glucose‐treated NP cells. The cells were transfected with LV‐NC or LV‐Atg7 before high‐glucose treatment (100 mM) or LV‐shHuR. A, B, The protein expression of Atg7 in rat NP cells with LV‐Atg7 transfection, detected by Western blot and quantified by Image J software. C, TEM images of autophagic vesicles in rat NP cells, with LV‐NC or LV‐Atg7 (scale bar: 0.5 μm). D, LC3‐II was detected by immunofluorescence combined with DAPI staining for nuclei (scale bar: 20 μm) E, F, The protein expression of p62 and the ratio of LC3‐II/ LC3‐I were detected by Western blot and quantified by Image J software. All data were shown as mean ± SD. *P < .05, **P < .01, &*P* < .001

As it is known from the study above that HuR regulates autophagy through Atg7, we next asked whether overexpression of Atg7 may reverse the detrimental effect of HuR deficiency in high glucose‐treated NP cells. We upregulated Atg7 in NP cells by lentivirus‐Atg7 (LV‐Atg7) transfection. Western blot analysis revealed that the Atg7 level was increased by LV‐Atg7, implying the successful establishment of Atg7‐upregulated NP cell model (Figure 5A‐B).

The TEM results revealed that the number of autophagosome vacuoles decreased in HuR‐down‐regulated NP cells, whereas such a change was attenuated by Atg7 upregulation, indicating that Atg7 reverses HuR‐down‐regulated autophagy in high glucose‐treated NP cells (Figure 5C). We also evaluated the autophagy by immunofluorescence and Western blot analysis and found that autophagy was inhibited by HuR downregulation, whereas Atg7 overexpression prompted the autophagy activation, as indicated by the LC3‐II expression (Figure 5D), the LC3‐II/ LC3‐I ratio and the p62 expression (Figure 5E‐F). These results suggest that Atg7 activates autophagy that is suppressed by HuR downregulation autophagy in high glucose‐treated NP cells.

### 3.8 Atg7 inhibits HuR‐regulated senescence in high glucose‐treated NP cells

**FIGURE 6:**
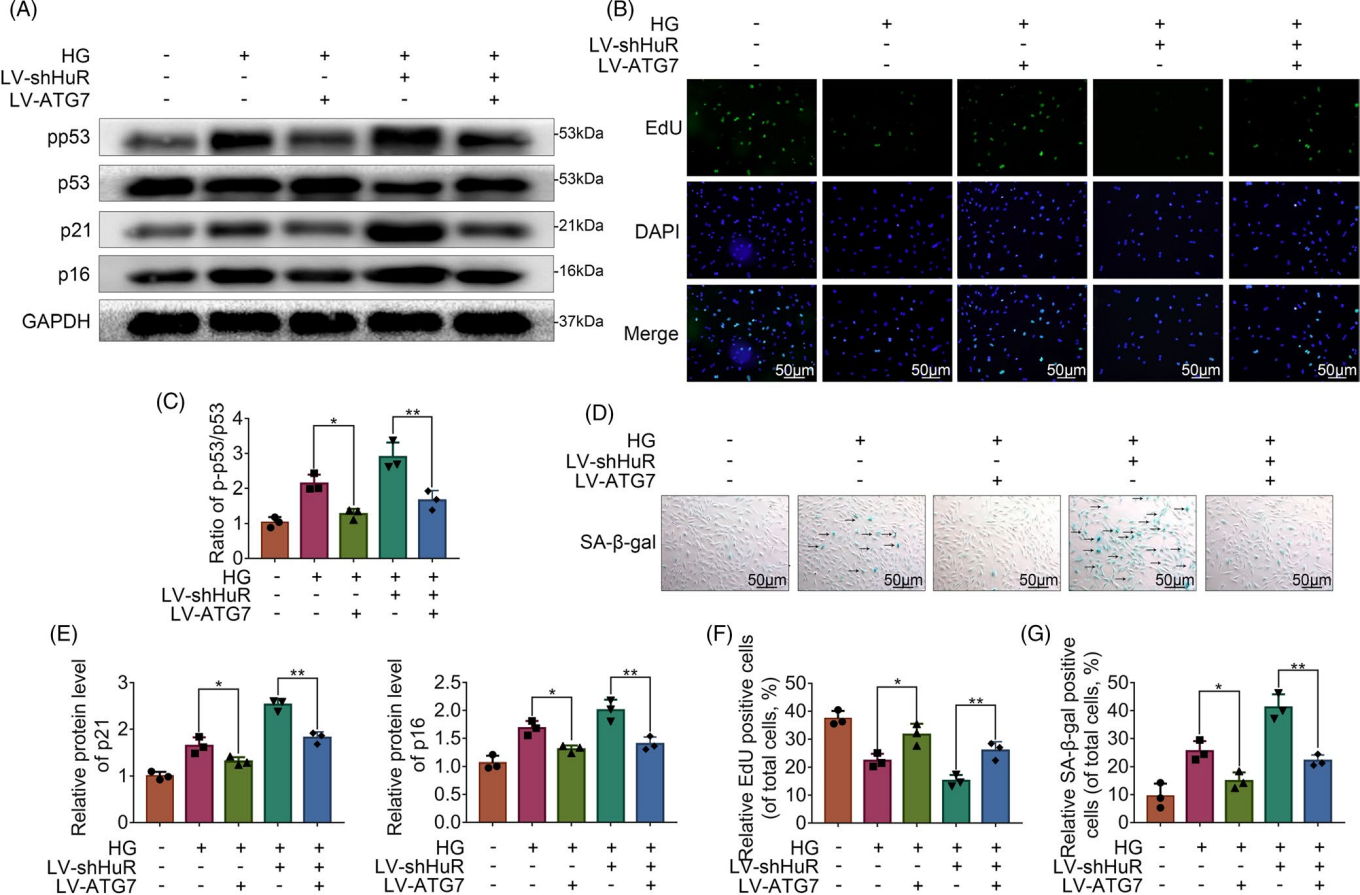
Atg7 inhibits HuR‐regulated senescence in high glucose‐treated NP cells. A, C, E, The protein expression of p21, p16INK4a and the ratio of p‐p53/p53, detected by Western blot. B, F, EdU staining for NP cells (scale bar: 50 μm). D, G, SA‐β‐gal staining for NP cells (scale bar: 50 μm). All data were shown as mean ± SD. *P < .05, **P < .01, ***P < .001

To further confirm whether Atg7 inhibits HuR‐regulated senescence in high glucose‐treated NP cells, we carried out the following experiments. The Western blot analysis showed that Atg7‐upregulation induced a significantly lower ratio of p‐p53/p53, and lower levels of p21 and p16INK4a in HuR‐down‐regulated NP cells (Figure 6A, C, E). The EdU assay revealed that the proliferative activity of NP cells was decreased in the HuR‐down‐regulated NP cells, while such a change was attenuated by Atg7 upregulation (Figure 6B, F). SA‐β‐gal assay revealed that HuR downregulation prompted SA‐β‐gal activity, whereas Atg7 upregulation inhibited the SA‐β‐gal activity (Figure 6D, G). Together, these results show that Atg7 may attenuate senescence that is induced by HuR deficiency in high glucose‐treated NP cells.

### 3.9 Overexpression of Atg7 ameliorates the progress of DB‐IVDD in vivo

To investigate the therapeutic effects of HuR and Atg7 on DB‐IVDD in vivo, we established an STZ‐induced diabetic rat model and injected lentivirus into the intervertebral disc to regulate the expressions of HuR and Atg7. The effect of the lentivirus is verified by histochemistry (Figure S6,S7).

Firstly, we explore the effect of HuR on DB‐IVDD in vivo. The results showed that HuR upregulation was not able to ameliorate the progress of DB‐IVDD in vivo, the phenomenon was addressed in the discussion. Alternatively, we examined the therapeutic potential of Atg7 on DB‐IVDD.

**FIGURE 7:**
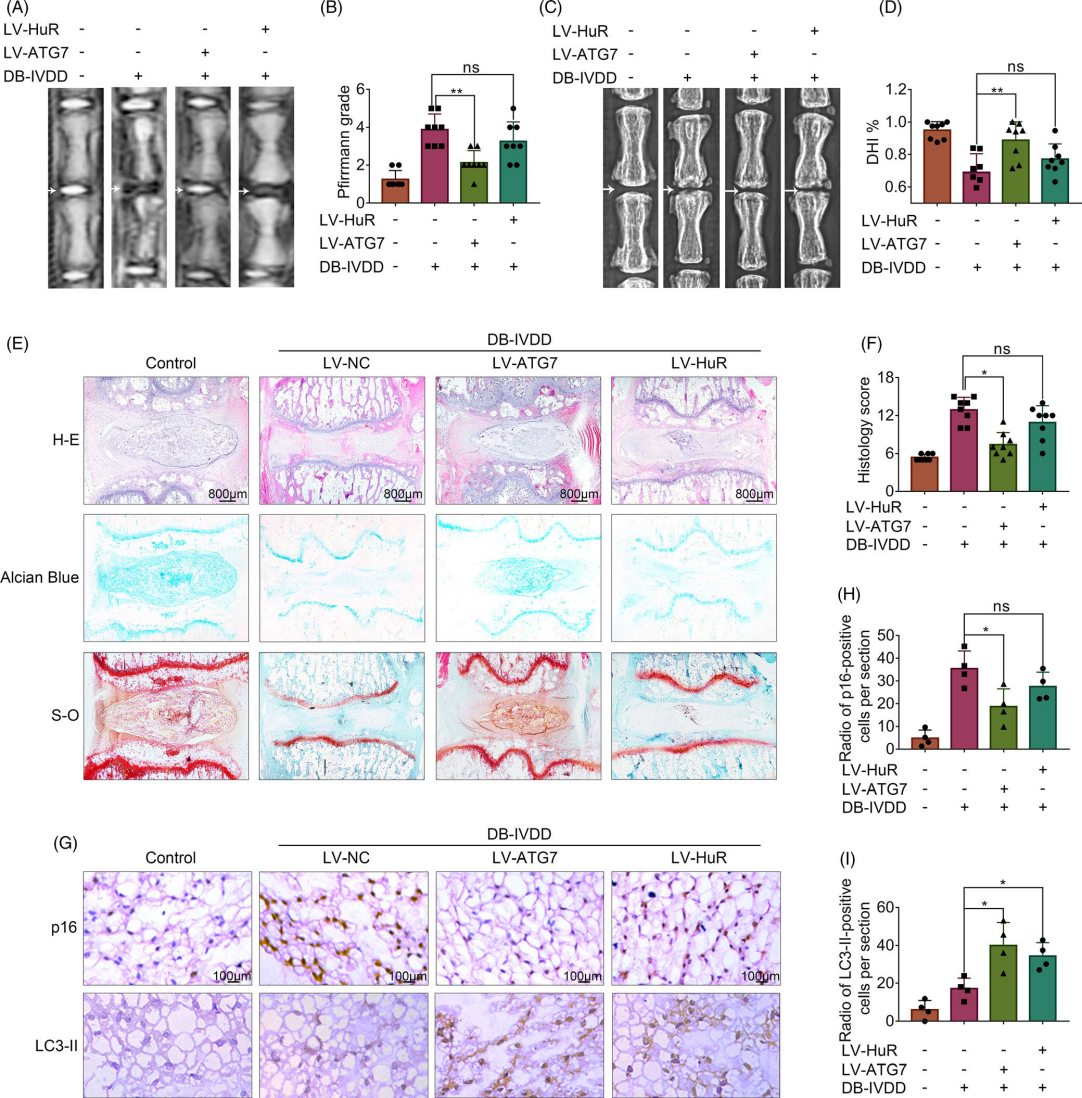
Overexpression of Atg7, instead of HuR, ameliorates the progress of DB‐IVDD in vivo. A, T2 weighted MRI of a rat‐tail disc at 3 weeks after disc puncture surgery, with or without lentivirus transfection (white arrows). B, The respective Pfirrmann grade scores of a rat‐tail disc. C, X‐ray of a rat‐tail disc at 4 weeks after disc puncture surgery in normal and diabetic rats, with or without lentivirus transfection (white arrows). D, The disc height index (DHI) of a rat‐tail disc. E, Representative H‐E, S‐O and Alcian Blue staining of NP tissues (scale bar: 800 μm). F, The histological grades evaluated in three groups. G‐I, The immunohistochemical staining of p16 and LC3‐II in intervertebral disc sections; the levels were determined using Image‐Pro Plus software. All data were shown as mean ± SD. *P < .05, **P < .01

We observed that MRI T2‐weight signal intensity of the intervertebral disc in the diabetic group was weaker than that in the control group, whereas such a change was reversed in the LV‐Atg7 group (Figure 7A). Subsequently, the Pfirrmann grade scores in the LV‐Atg7 group were higher, compared with scores in the diabetic group (Figure 7B). Additionally, the X‐ray results also showed that disc height of the intervertebral disc in the diabetic group was lower than that in the control group, whereas such a change was reversed in the LV‐Atg7 group (Figure 7C‐D).

Through H‐E staining, we found that the number of NP cells was decreased and they were replaced by the fibrochondrocytes, and the cartilaginous endplate was collapsed in the diabetic group, whereas Atg7 upregulation ameliorated these degenerations (Figure 7E‐F). As for Alcian Blue and S‐O staining, which is sensitive to proteoglycan and hyaluronic acid, we found the colour reaction significantly decreased in the diabetic group, whereas such a change was ameliorated in the LV‐Atg7 group (Figure 7E).

Further, we observed that senescence was prompted in the diabetic group, as indicated by the expression of p16INK (detected by immunohistochemistry, Figure 7G‐H), whereas Atg7 upregulation ameliorated the promoted senescence. In addition, we examined the expression of LC3‐II by immunohistochemistry, and the results showed that the levels of LC3‐II were prompted in the diabetic group, whereas Atg7 upregulation further prompted the expression of LC3‐II (Figure 7G, I).

## 4 DISCUSSION

HuR is an RNA‐binding protein, which selectively binds to AU‐rich elements of mRNAs.^26^ HuR stabilizes mRNA to regulate gene expression, which in turn affects various biological progresses. Zhang *et al* found that HuR promoted breast cancer cell proliferation by binding to CDK3 mRNA,^31^ and Liu *et al* found that HuR enhanced early restitution of the intestinal epithelium by increasing Cdc42 translation.^32^ In addition, Pan *et al* found that HuR stabilized mRNA to adjust pH homeostasis and extracellular matrix gene expression in NP cells under hypoxia ^33^; Mynatt *et al* also found that HuR affected the expressions of genes that involve in oxidative phosphorylation and mitochondrial fatty acid oxidation, to regulate the metabolism of diabetic mice.^27^ Therefore, we assume that HuR can play a role in the progress of DB‐IVDD. Indeed, in this study, we found that the expression of HuR was decreased in diabetic NP tissues and high glucose‐treated NP cells.

Accumulating evidence demonstrated that increased senescence of NP cells is one of the inducers of IVDD.^34^ Previous researches also demonstrated that inhibiting senescence of NP cells is regarded as an effective therapeutic strategy of IVDD.^35^ In this study, we found that HuR downregulation induced increased senescence in high glucose‐treated NP cells. In line with our results, Wang *et al* reported that the loss of HuR was linked to senescence of fibroblasts ^36^; Lee *et al* demonstrated that the loss of HuR facilitated cellular senescence by post‐transcriptional regulation of TIN2 mRNA in HeLa cells ^37^; Hashimoto *et al* also demonstrated that a reduction in HuR expression exhibited multiple senescence‐associated phenotypes *via* activating NF‐kappaB signalling pathway.^38^ Although HuR has been illustrated to be negatively related to senescence, its mechanism remains unclear.

Studies have demonstrated that autophagy plays a protective role in diabetes and IVDD ^39^; also, autophagy has been shown to combat against senescence.^25^ Based on in vitro investigation, we found that HuR regulates senescence through autophagy. Corresponding to our results, other studies have demonstrated that HuR affects a variety of biological progresses through regulating autophagy. For example, Zili *et al* found that HuR promoted autophagy *via* Beclin 1 ^26^; Viiri *et al* also found that HuR relieved macular degeneration by activating autophagy *via* p62 ^40^; and Li *et al* found HuR modulated autophagy to defence against invasive pathogens in the intestinal epithelium *via* Atg16L1.^41^

The mechanistic study revealed that HuR regulates autophagy through Atg7. Atg 7 is an autophagy‐related protein, it may not only conjugate to Atg5 and Atg12, but also conjugate to Atg8, which further forms protein complex with these proteins and involve in autophagosome membrane formation.^42^ Lee *et al* showed in Atg7‐deficient mice that the loss of Atg7 may lead to autophagy insufficiency and induce diabetes in mice ^43^; Zhang *et al* also found that suppression of Atg7‐dependent autophagy promoted senescence in melanocytes.^44^ These studies suggest Atg7 to be protective in diseases. In line with these studies, we found that upregulation of Atg7 may suppress senescence in high glucose‐treated NP cells.

Regarding the function of HuR in NP cells, we attempted to rescue high glucose‐induced senescence *via* manipulating HuR. Although downregulation of HuR leads to increased senescence in high glucose‐treated NP cells, HuR upregulation is not able to significantly inhibit the increased senescence.

The possible reason is that HuR may have pleiotropic roles on senescence through interacting with different mRNA subsets (Figure S8). Galban *et al* reported that HuR‐regulated *TP53* mRNA expression in renal carcinoma cells ^45^; Wang *et al* showed HuR‐regulated p21 expression via *CDKN1A* mRNA stabilization in colorectal carcinoma cells ^46^; and Pang *et al* implied that HuR regulated the expression of p16 *via* binding to mRNA in HeLa cells.^47^ p53, p21 and p16 are known to be the key factors that may promote senescence. Although our study revealed that HuR can promote autophagy through Atg7 to prevent senescence, HuR overexpression may also directly induce senescence through upregulating the mRNA expression of p53, p21 and p16 (Figure S9). Thus, HuR may not be suitable to be a target for senescence or IVDD. Alternatively, we examined the therapeutic potential of Atg7, the HuR‐regulated protein. And we demonstrated that that Atg7 overexpression can effectively inhibit senescence in high glucose‐treated rat NP cells. Upregulation of Atg7 prompted autophagy activity and inhibited senescence in HuR‐downregulated NP cells. Meanwhile, Atg7 overexpression ameliorated the progress of DB‐IVDD in vivo.

**FIGURE 8:**
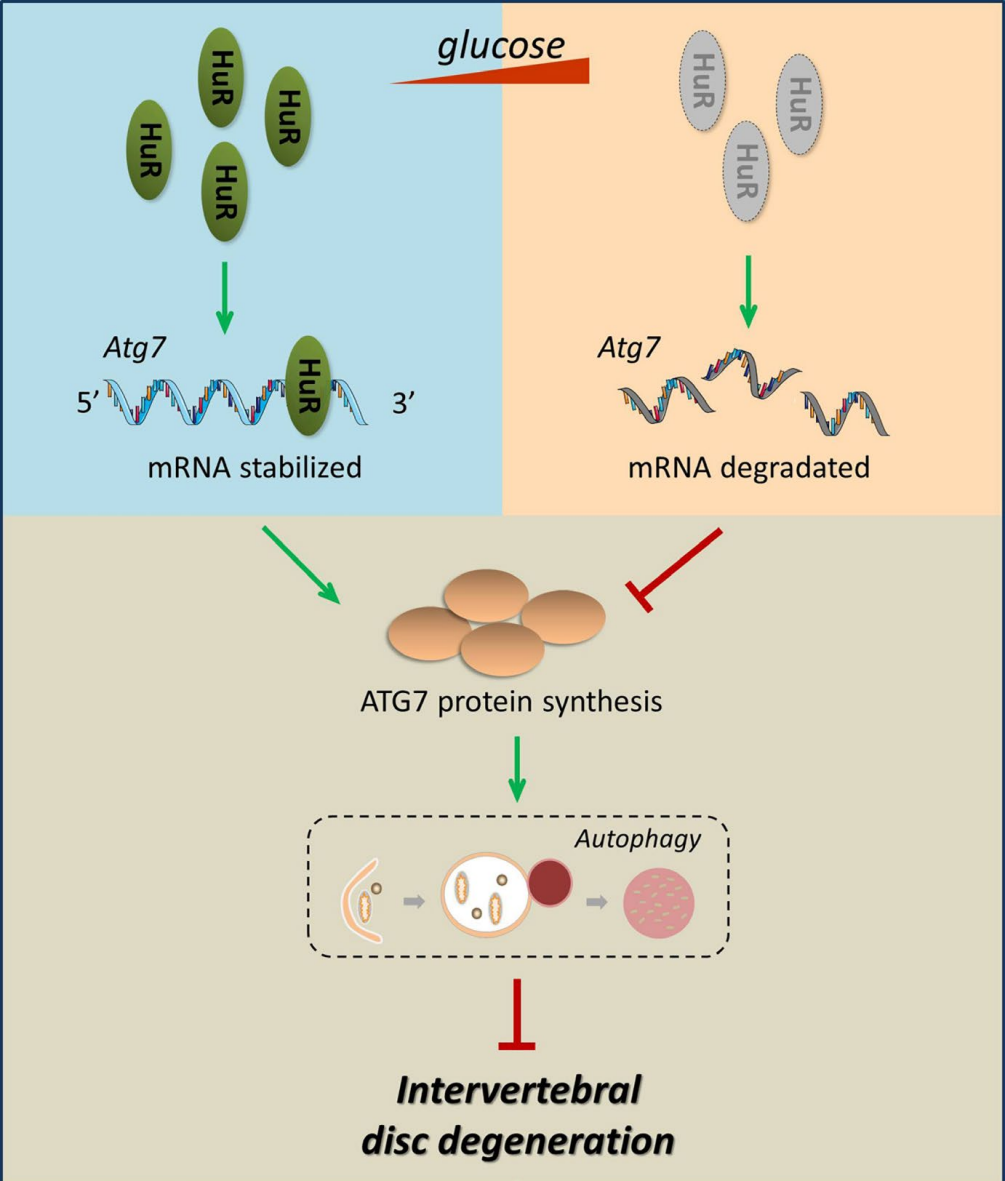
Schematic illustration. With high‐glucose treatment, the expression of HuR is decreased, which induces autophagy inactivation and increased senescence. Mechanistic study reveals that HuR regulates Atg7 expression through binding to Atg7 AU‐rich element and adjusting its mRNA stability

In conclusion, we demonstrate that HuR downregulation prompts senescence through autophagy inactivation in high glucose‐treated NP cells. Mechanistic study reveals that HuR regulates Atg7 expression through binding to Atg7 AU‐rich element and adjusting its mRNA stability (Figure 8). Moreover, Atg7 inhibits HuR‐regulated senescence in high glucose‐treated NP cells, and Atg7 overexpression ameliorated the progress of DB‐IVDD in vivo, which suggests Atg7 to be a potential therapeutic target for DB‐IVDD.

## CONFLICT OF INTEREST

There is no conflict of interest between the authors.

## AUTHOR CONTRIBUTIONS

XZ, YZ and XW designed the experiments, and revised the manuscript before submitting. ZS, TX, ZM, ZP, NT, YW, LS and AW performed the experiments, acquired data and drafted the article. LN, ZP and LC analysed and interpreted the data. SH and WG provided reagents and materials tools. ZS collected relevant papers in this field.

## Supporting information

Figure S1‐S9

Supplementary Material

## Data Availability

Some of all data are available from Supplemental files.
