# Supplementary material for: RNA‐binding protein HuR suppresses senescence through Atg7 mediated autophagy activation in diabetic intervertebral disc degeneration
Source: Cell Prolif. 2020 Dec 28;54(2):e12975. doi: 10.1111/cpr.12975 (PMC7848958; doi:10.1111/cpr.12975)
Supplement: Supplementary file 1 — Figure S1‐S9 [file CPR-54-e12975-s001.docx]

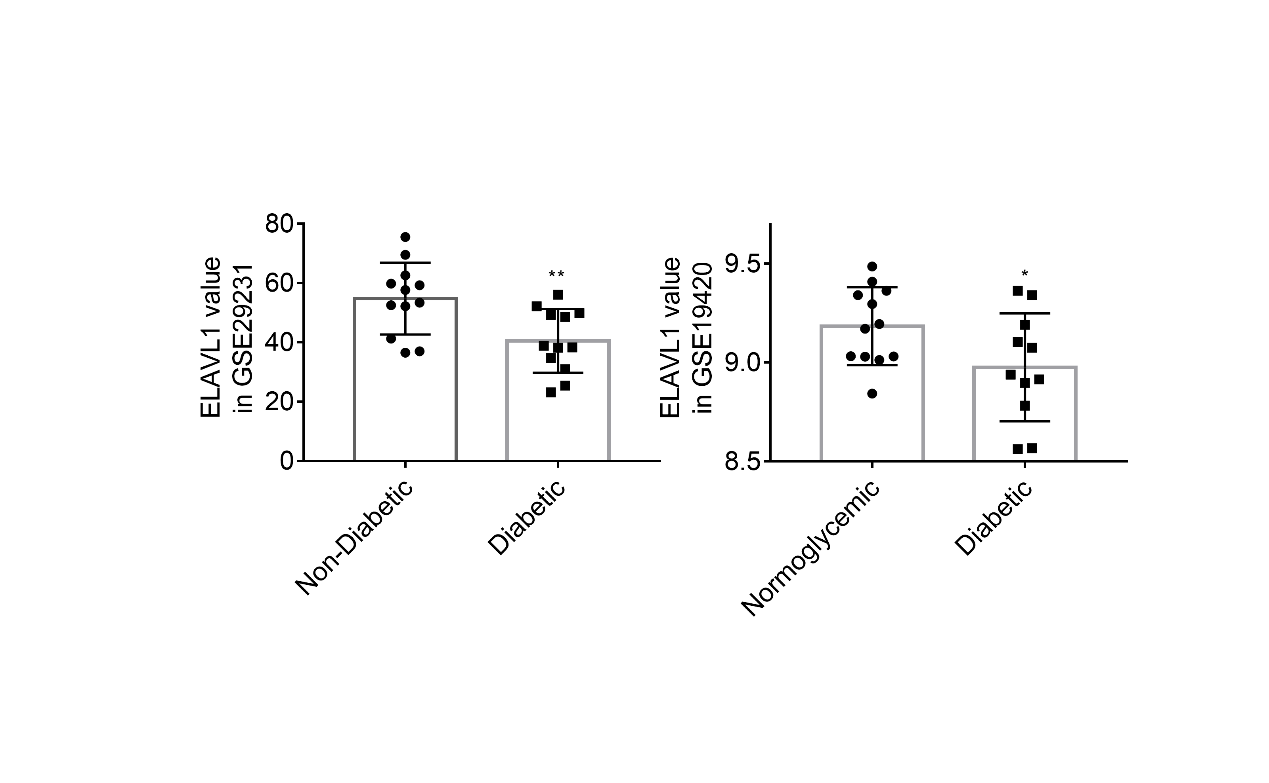


**Figure S1.**

The HuR gene expression from different diabetic tissues (GSE29231 and GSE19420). All data were shown as mean ± SD. **p*<0.05, ***p*<0.01.


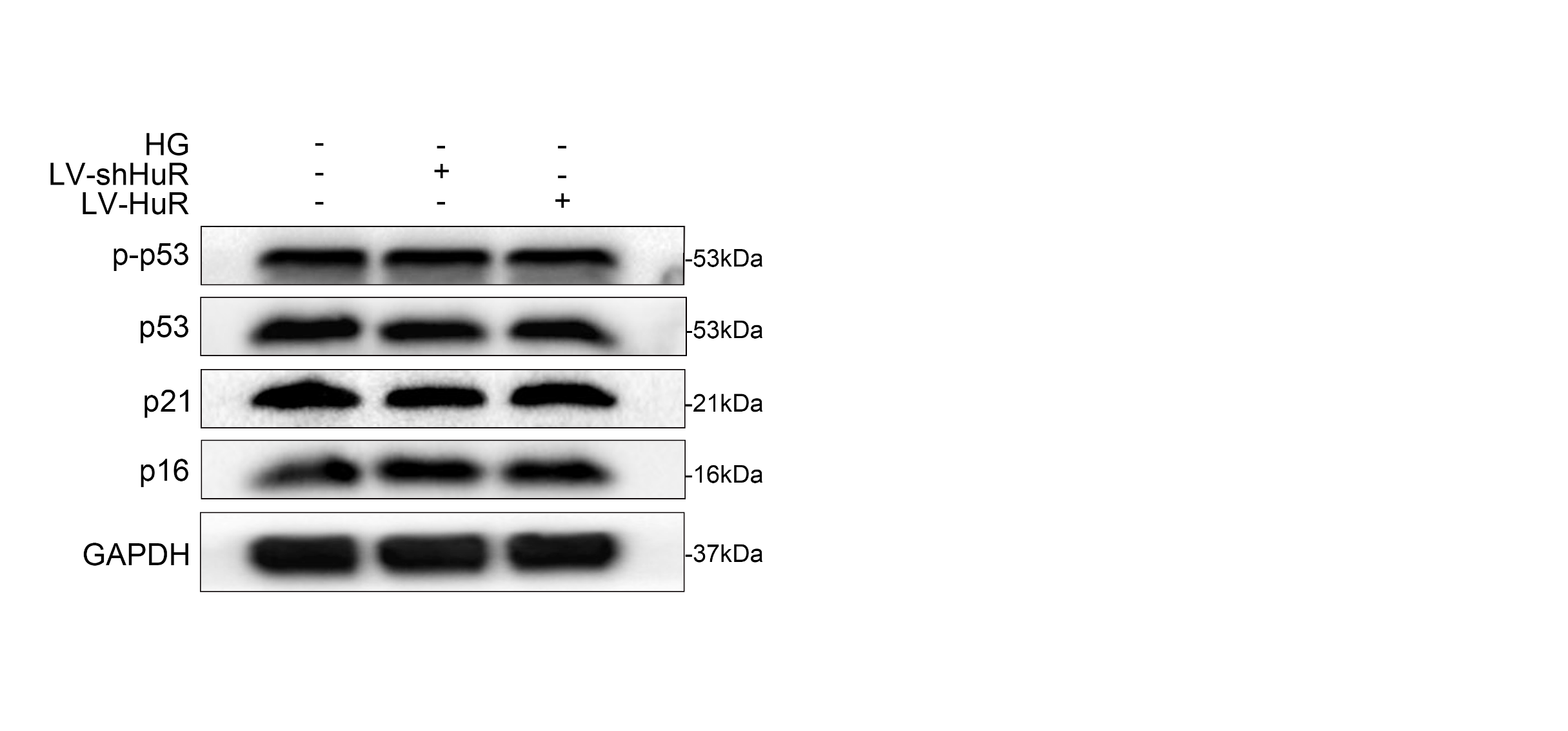


**Figure S2.**

Without high-glucose treatment, the protein expressions of p-p53, p53, p21 and p16 in rat NP cells.

**
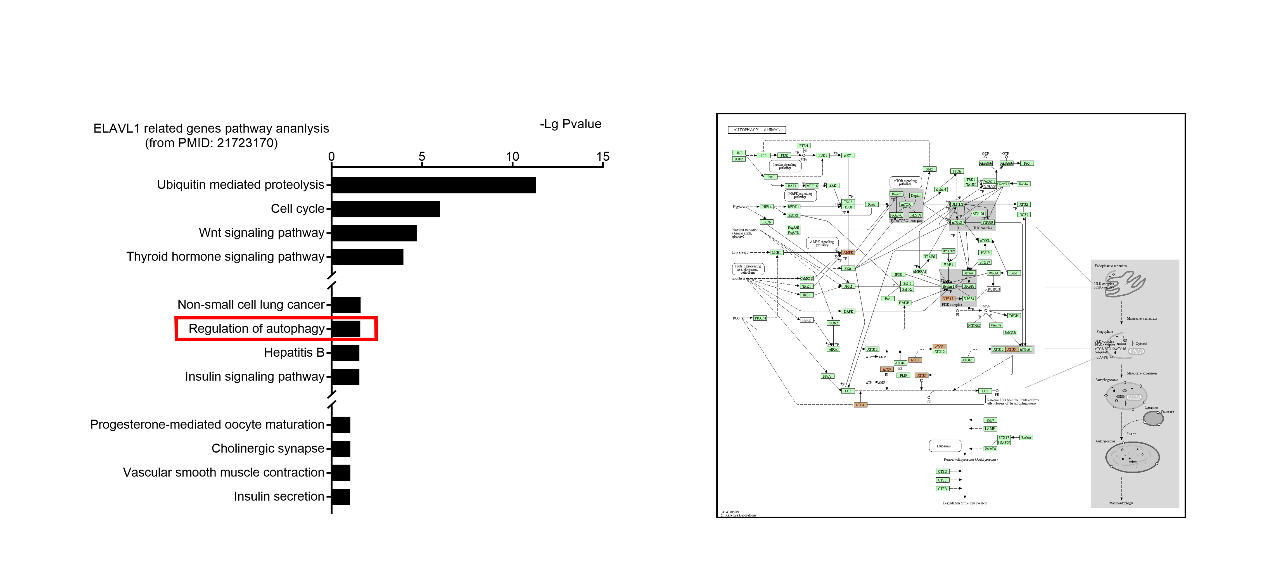
**

**Figure S3.**

KEGG pathway analysis of HuR-regulated genes (top 2500), and data from PMID 217231170.


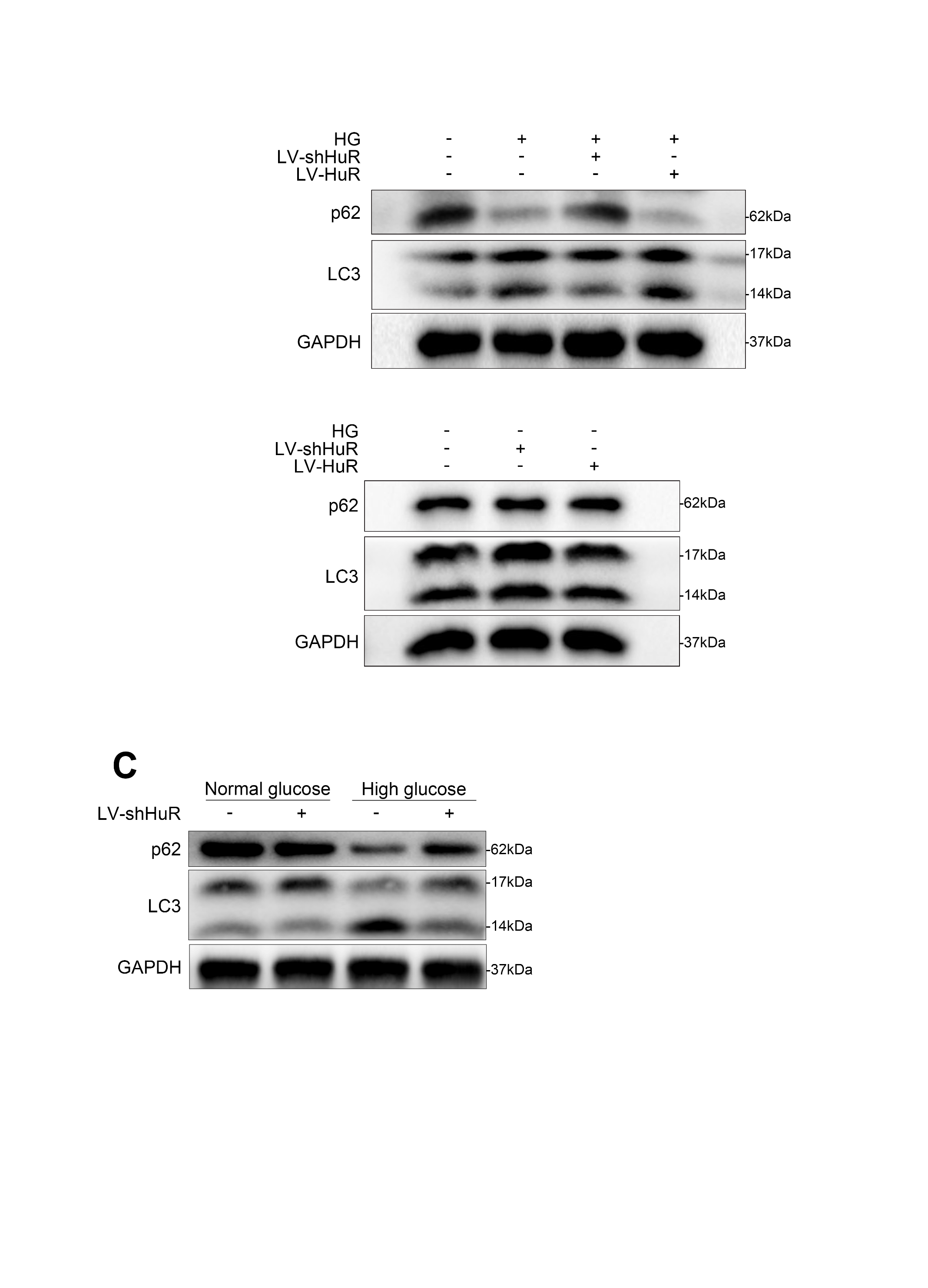


**Figure S4.**

Without high-glucose treatment, the protein expressions of p62 and LC3 in rat NP cells.

**
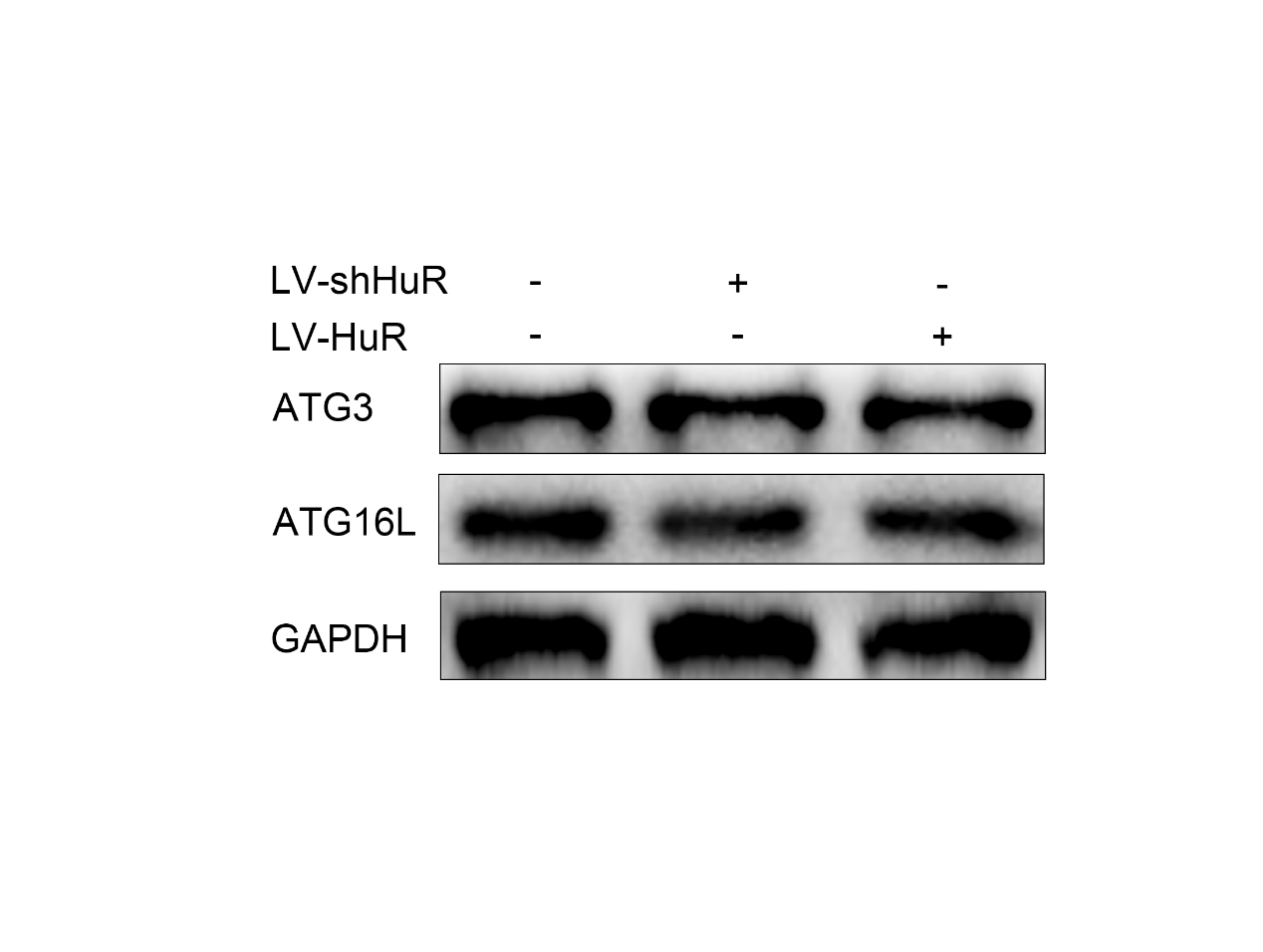
**

**Figure S5.**

The protein expressions of ATG3 and ATG16 with high-glucose treatment in rat NP cells.


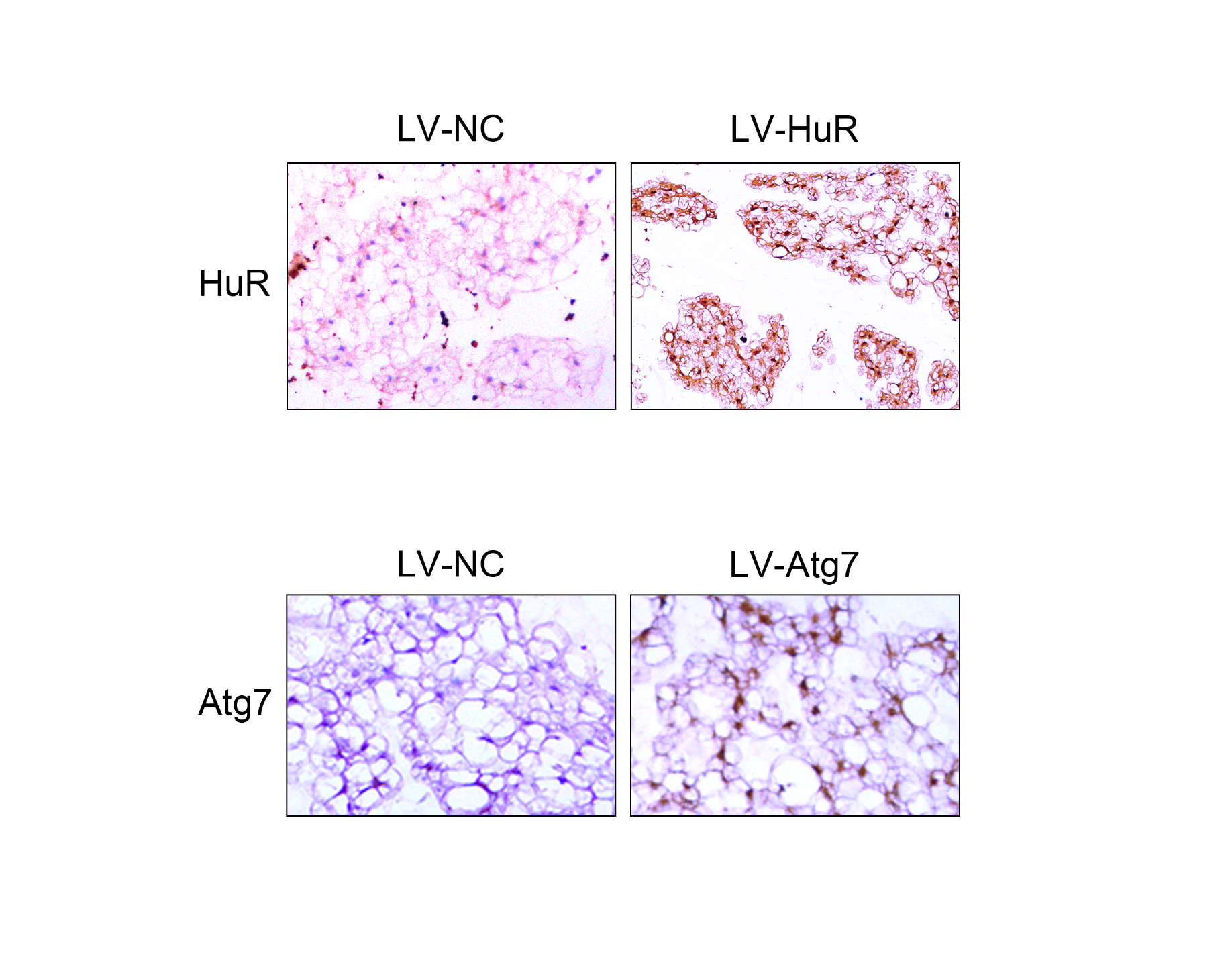


**Figure S6.**

The immunohistochemical staining of HuR in intervertebral disc sections.


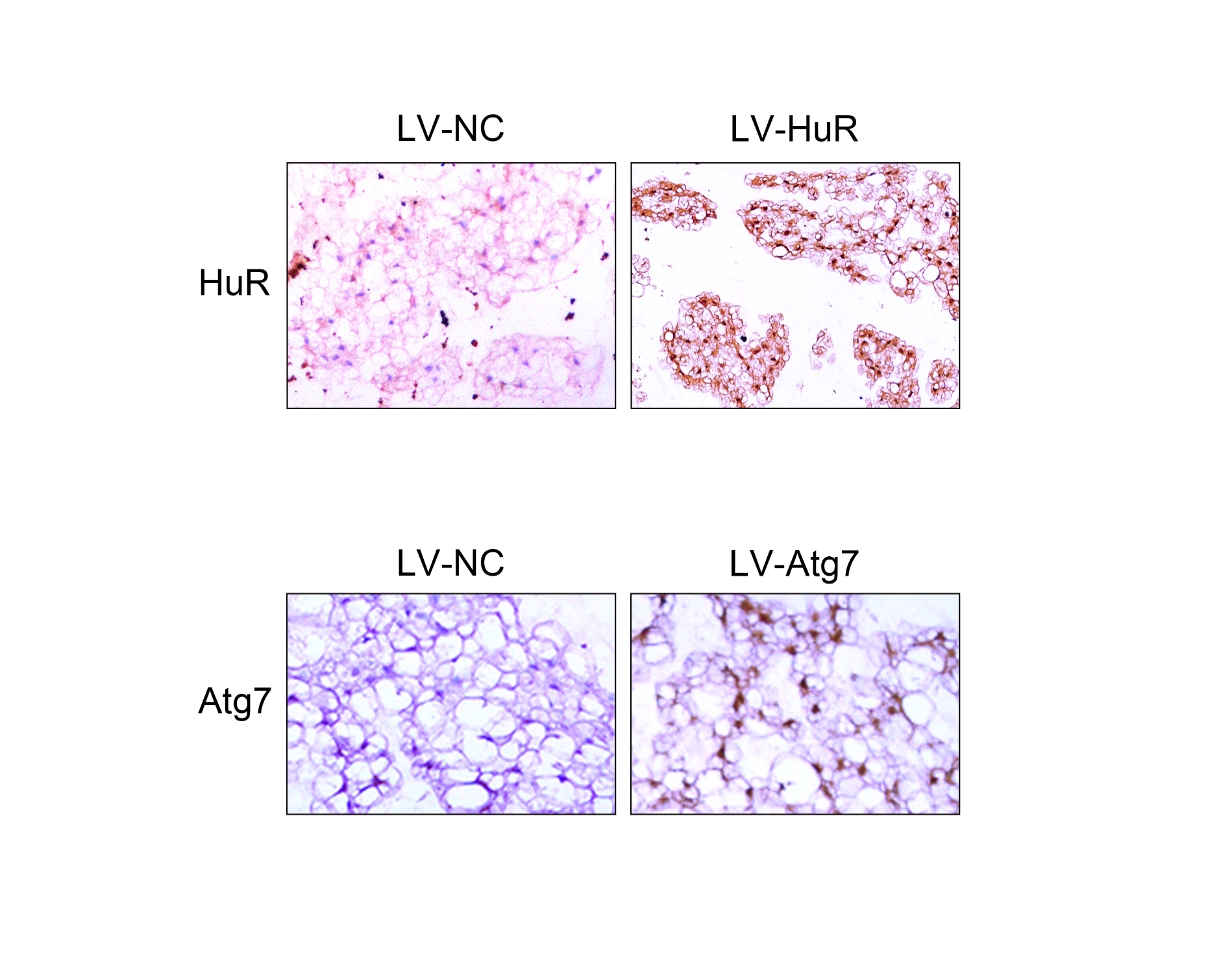


**Figure S7.**

The immunohistochemical staining of Atg7 in intervertebral disc sections.


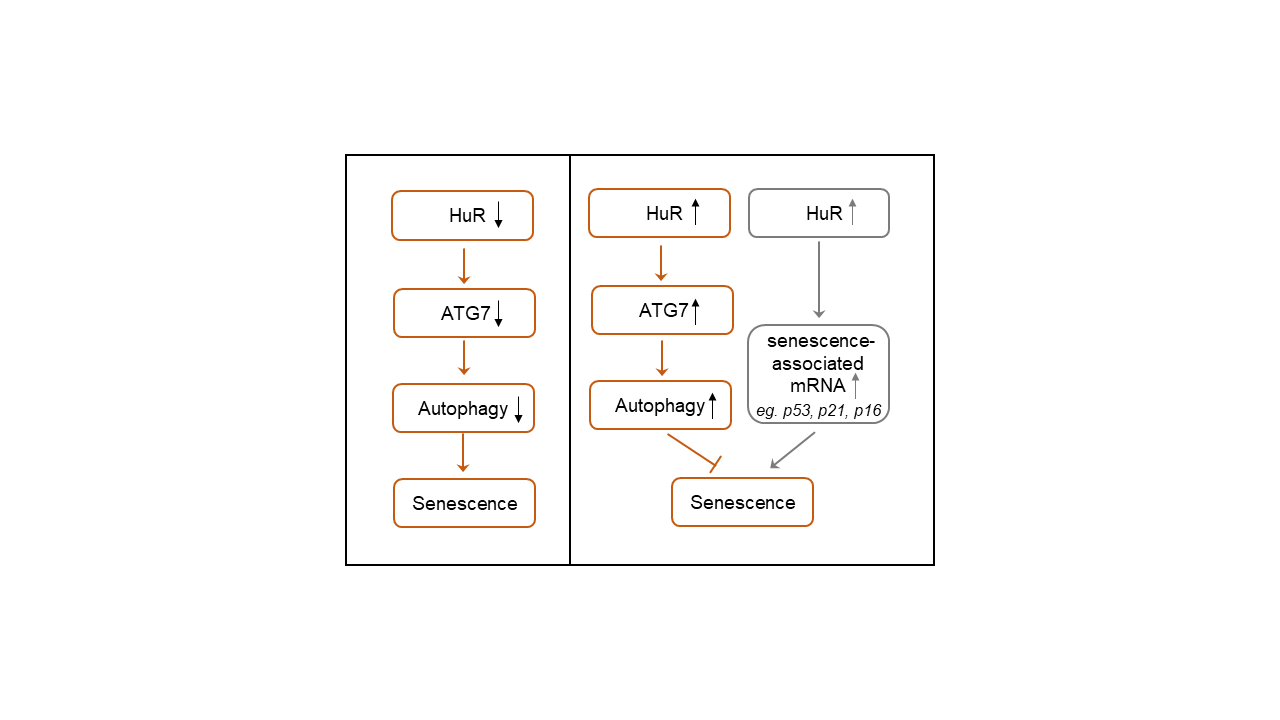


**Figure S8.**

HuR may have pleiotropic roles on senescence through interacting with different mRNA subsets

When the expression of HuR increased, HuR not only promotes autophagy through Atg7 to prevent against senescence, but also stabilizes senescence-associated mRNA (*eg.* p53, p21 and p16) to prompt senescence. Thus, HuR may not be suitable to be a direct target for senescence.


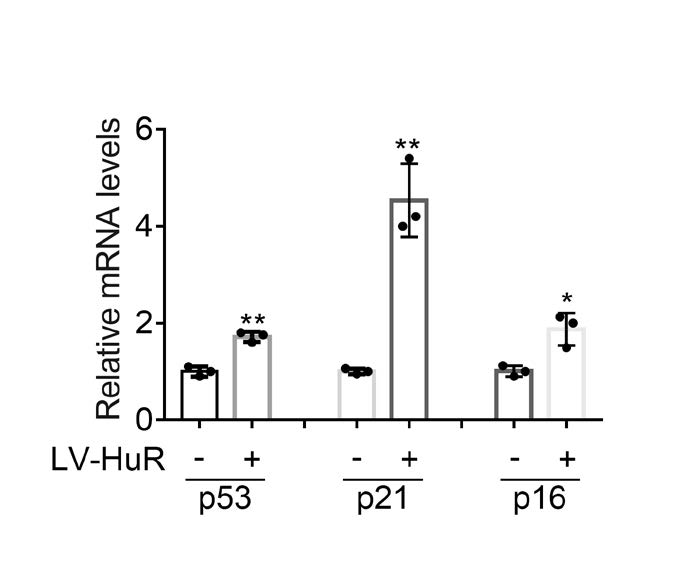


**Figure S9.**

The gene expressions of p53, p21, and p16, after transfecting with LV-HuR lentivirus. All data were shown as mean ± SD. *p<0.05, **p<0.01.
