# Supplementary material for: RNA‐binding protein HuR suppresses senescence through Atg7 mediated autophagy activation in diabetic intervertebral disc degeneration
Source: Cell Prolif. 2020 Dec 28;54(2):e12975. doi: 10.1111/cpr.12975 (PMC7848958; doi:10.1111/cpr.12975)
Supplement: Supplementary file 2 — Supplementary Material [file CPR-54-e12975-s002.docx]

## Supplemental Material and methods

### *Cell viability assay*

According to the manufacturer's protocol, cell viability was detected using the CCK-8. The NP cells were cultured for 24 hours in 96-well plates (50,000 cell/cm^2^) and then treated correspondently. At the indicated time point, the cells were washed with PBS, followed by the addition of 100 μl of DMEM/F12 containing 10 μl of CCK-8 solution into each well of the plate, and incubated for another 1 hour at 37°C. The absorbance of the wells was measured using a microplate reader (Thermo, Rockford, USA) at 450 nm.

### *Real-time PCR*

After treatment, total RNAs were extracted from NP cells by using TRIzol reagent (Invitrogen). An aliquot of 1,000 ng of the total RNA was reversely transcribed into cDNA (MBI Fermantas, Germany) using the PrimeScript-RT reagent kit (Takara, Japan) based on the CFX96 Real-Time PCR System (Bio-Rad Laboratories, CA). Amplification of the cDNA was performed using SYBR Premix Ex Taq based on the CFX96 Real-Time PCR System (Bio-Rad Laboratories, CA). The cycle threshold (Ct) values were determined and normalized to the level of a housekeeping gene (*GAPDH*). The expression of the target genes in different groups was evaluated using the 2^−ΔΔCt^ method. The forward and reverse primer sequences are provided in Table S1.

**Supplemental Table 1 Primers used for mRNA expression studies using PCR**

| Gene | Forward primer (5'->3') | Reverse primer (3'->5') |
| --- | --- | --- |
| ATG1-ULK1 | CCCAGCAACATCCGAGTCAAGA | CAGGTCAGCCTTCCCATCGTAGT |
| ATG2 | CCTTTGGGTCATCCACAGCC | ATGCCTTAGCCATGCCTTCC |
| ATG3 | CGGCTCTGGCTGTTTGGCTAT | TGGTGGGAGGTGAGGATGGTT |
| ATG4 | CTGCTTTATCCCCGACGAGA | AGGCTGCTGTTCCACCAACT |
| ATG5 | ATTCCAACGTGCTTTACTCTCTATC | AAACCAAATCTCACTAACATCTTCT |
| ATG6-BECN | AATCTTGCCTTTCTCCAC | TTGCCGTTGTACTGTTCT |
| ATG7 | TGGGAAGCCATAAAGTCAGG | GGCAGCAAAACCAGTAGTAGAA |
| ATG8-LC3 | CATCTTGCCCCTCATCCACCT | GAGGAAATGACCACAGATCCACATA |
| ATG9 | CTTGGCACCACATTGAAAACC | AAGGCAACCACAAAGAGGAAC |
| ATG10 | AGACTCCAACACCACATCGG | ACGGCTTCAGAATCATCCAC |
| ATG12 | GGCACCAGCTCTAGGCTTATAGTTG | GTTGTTCCACAGCATTTTCCATG |
| ATG13 | TGGACTTGGGGACCTTTTAC | GTCTTCTGCCATAGACTGAGCT |
| ATG14 | CCTGGAGTTGCTGGGGAATC | GGTGCTCTGGCTCTGGGAAA |
| ATG16 | CTACAGGCTGAAAAGCACGAC | CCAACTGAGCTAACTCCCCAC |
| p62 | TCCTCCTTGGCTTTGTCT | CTGAAAGAGCGGGTACTGA |
| ELAVL1 | GGGCCTCCAAACCTCCTA | GGGCGAATCATCAACTCC |

### *Western blot (WB) analysis*

Proteins from NP cells were lysed in radioimmunoprecipitation assay (RIPA) buffer (Beyotime) with 1 mM phenylmethanesulfonyl fluoride (PMSF) (Beyotime), and then centrifuged for 15 min at 12,000 rpm and 4°C. The protein concentration was measured using the BCA protein assay kit (Beyotime). An aliquot of 40 ng of protein was separated via 8–12% (w/v) sodium dodecyl sulfate polyacrylamide gel electrophoresis and blotted onto a polyvinylidene ﬂuoride membranes (Bio-Rad, Hercules, CA, USA). After blocking with 5% non-fat milk for 2 hours, the membranes were incubated with primary antibodies against HuR (1:1000), Atg7 (1:1000), p21WAF1 (1:800), p62 (1:1000), LC3 (1:1000), p16INK4A (1:800), p53 (1:1000), p-p53 (1:700), and GAPDH (1:3000) overnight at 4°C, and then subsequently incubated with the respective secondary antibodies for 2 hours at room temperature. After washing three times with Tris-buffered saline with Tween® 20, the blots were visualized using the electrochemiluminescence plus reagent (Invitrogen). The signals were visualized based on the ChemiDicTM XRS + Imaging System (Bio-Rad, Hercules, CA, USA), and the band density was quantified with Image Lab 3.0 software (Bio-Rad, Hercules, CA, USA).

### *Immunofluorescence*

NP cells were plated in a 6-well plate. After treatment, the NP cells were washed with ice-cold PBS, fixed with 4% (v/v) paraformaldehyde for 15 min, and permeated using 0.1% Triton X-100 diluted in PBS for 10 min. Then, the cells were blocked with 5% bovine serum albumin for 1 hour at 37°C, followed by incubating with a primary antibody overnight at 4°C. At the next day, the cells were incubated with Alexa Fluor® 488- or 94-conjugated secondary antibody (1:300) for 1 hour at room temperature and labelled with 4′,6-diamidino-2-phenylindole (DAPI) for 5 min. Finally, three fields of view from each slide were randomly chosen for microscopic observation under the Nikon ECLIPSE Ti microscope (Nikon, Japan).

## *EdU staining*

NP cell proliferation was evaluated using the Click-iT EdU microplate assay kit (Invitrogen), by measuring the uptake of 5-ethynyl-2’-deoxyuridine (EdU) into DNA. According to the manufacturer's instructions, NP cells were labelled with EdU that was coupled with Oregon Green azide, after the incubation. Next, EdU incorporated into the DNA was detected using HRP-conjugated anti-Oregon Green antibody and Amplex UltraRed. Finally, the NP cells were observed under a fluorescence microscope (Olympus Inc.).

## *SA-β-gal staining*

NP cell senescence was evaluated using the senescence-associated β-galactosidase (SA-β-gal) staining kit (Beyotime), by measuring the upward of β-galactosidase in the nucleus. According to the manufacturer's instructions, NP cells on plates were fixed with 0.2% glutaraldehyde for 10 min at room temperature. The cells were stained with X-gal staining solution overnight at pH 6.0. Images were captured using the Olympus IX71 microscope, and the percentages of SA-β-gal-positive cells were quantified for statistical analysis.

## *Transmission electron microscopy*

After ﬁxation in 2.5% (w/v) glutaraldehyde overnight and post-ﬁxed in 2% (w/v) osmium tetroxide, the NP cells were stained with 2% (w/v) uranyl acetate and dehydrated in acetone. Images were captured with the aid of a Hitachi transmission electron microscope (Hitachi, Tokyo, Japan).

### *Histopathological analysis*

The rats were euthanized by an intraperitoneal over-dosed 4% pentobarbital, and the tails were collected. The specimens were decalcified, fixed in formaldehyde, dehydrated, and embedded in paraffin. The tissues were cut into 5-μm sections, stained with haematoxylin and eosin (H&E), Safranin O-fast green (S-O), or Alcian Blue. The cellularity and morphology of the intervertebral disc were examined by a separate group of experienced histological researchers in a blinded manner under a microscope (Olympus Inc., Tokyo, Japan).
